# The Insulin-Mediated Modulation of Visually Evoked Magnetic Fields Is Reduced in Obese Subjects

**DOI:** 10.1371/journal.pone.0019482

**Published:** 2011-05-11

**Authors:** Martina Guthoff, Krunoslav T. Stingl, Otto Tschritter, Maja Rogic, Martin Heni, Katarina Stingl, Manfred Hallschmid, Hans-Ulrich Häring, Andreas Fritsche, Hubert Preissl, Anita M. Hennige

**Affiliations:** 1 Internal Medicine IV, Department of Endocrinology and Diabetes, Angiology, Nephrology and Clinical Chemistry, University Hospital, University of Tübingen, Tübingen, Germany; 2 MEG Center, University of Tübingen, Tübingen, Germany; 3 Center for Ophthalmology, University of Tübingen, Tübingen, Germany; 4 Department of Neuroendocrinology, University of Lübeck, Lübeck, Germany; 5 Department for Obstetrics and Gynecology, Medical College, University of Arkansas, Little Rock, Arkansas, United States of America; University of Las Palmas de Gran Canaria, Spain

## Abstract

**Background:**

Insulin is an anorexigenic hormone that contributes to the termination of food intake in the postprandial state. An alteration in insulin action in the brain, named “cerebral insulin resistance”, is responsible for overeating and the development of obesity.

**Methodology/Principal Findings:**

To analyze the direct effect of insulin on food-related neuronal activity we tested 10 lean and 10 obese subjects. We conducted a magnetencephalography study during a visual working memory task in both the basal state and after applying insulin or placebo spray intranasally to bypass the blood brain barrier. Food and non-food pictures were presented and subjects had to determine whether or not two consecutive pictures belonged to the same category.

Intranasal insulin displayed no effect on blood glucose, insulin or C-peptide concentrations in the periphery; however, it led to an increase in the components of evoked fields related to identification and categorization of pictures (at around 170 ms post stimuli in the visual ventral stream) in lean subjects when food pictures were presented. In contrast, insulin did not modulate food-related brain activity in obese subjects.

**Conclusions/Significance:**

We demonstrated that intranasal insulin increases the cerebral processing of food pictures in lean whereas this was absent in obese subjects. This study further substantiates the presence of a “cerebral insulin resistance” in obese subjects and might be relevant in the pathogenesis of obesity.

## Introduction

Obesity with its growing incidence and prevalence and its costs for the health system is currently a research focus in different scientific disciplines ranging from internal medicine to neuroscience. However, the underlying mechanisms that lead to overeating and obesity are not yet fully understood.

Human eating behavior is controlled by homeostatic and hedonic mechanisms [1], [2]. Whereas homeostatic mechanisms are based on signals from the periphery to the brain [3], [4], the hedonic aspects are based on emotional factors which are triggered by visual, olfactory and gustatory signals [5], [6].

In the homeostatic regulation of food intake, the modulation is basically achieved by nutritional and hormonal factors. One of the most prominent hormons in the postprandial state is insulin, which is secreted from beta-cells after a meal. Insulin receptors are widely expressed throughout the brain, especially in the hypothalamus, the olfactory bulb, the cerebellum and the cortex [7]–[9]. Various animal studies demonstrated that insulin in the brain controls and suppresses food intake [10]–[12]. Furthermore, knock-out models for insulin receptors in the brain displayed obesity and insulin resistance [13]. In humans, we demonstrated in a magnetoencephalographic (MEG) study that the insulin-mediated increase in spontaneous brain activity and auditory evoked fields during a hyperinsulinemic-euglycemic clamp is reduced in obese subjects [14]. During visual stimulation with a checkerboard, Benedict et al. [15] and Seaquist et al. [16] showed no effect of insulin on visual evoked potentials. However, Benedict et al. reported an effect of insulin on the later evoked component (P300) in a recognition memory task and they further demonstrated an improvement in memory by insulin [17].

Most of the studies in humans to date were performed with systemic insulin application and alterations at the blood-brain barrier in obese subjects were not taken into account [18]. Therefore, in the present study, insulin was administered intranasally to enter the brain via the olfactory nerve and bulb and to raise insulin concentrations in the cerebrospinal fluid without relevant absorption to the systemic blood circulation [19]–[21]. In this respect, intranasal insulin is a useful tool to determine the effect of insulin in the brain without affecting glucose metabolism in the periphery. We recently used this study protocol in a functional magnetic resonance imaging study (fMRI) to investigate the effect of insulin on neuronal activity and detected a reduction in the blood oxygen level dependent response (BOLD) after intranasal administration of insulin compared to placebo in the fusiform gyrus [22].

There have been few studies to date dealing with the neuronal processes related to food pictures. One group by Porubska et al. showed in a fMRI study that the BOLD response was increased by food pictures compared to non-food pictures [23]. In addition, studies performed with EEG and MEG have demonstrated a very detailed insight into the time dynamics of processing food-related pictures in the human brain [23]–[26]. In our previous study with MEG [24] we were able to show the temporal sequence of neuronal activation during visual processing of food pictures, whereas in the current study we have focused on the effect of insulin in this network.

## Methods

### Objectives

We hypothesized that insulin modulates food specific activation in the brain dependent on body weight, and therefore evaluated evoked magnetic fields in lean and obese subjects before and after intranasal insulin administration.

### Participants

Subjects were recruited via email advertisement. We originally included 26 healthy subjects in the study. Six subjects had to be excluded based on the following criteria: one subject due to a severe migraine occured during the study, another subject did not show up for the second recording session, two further subjects were excluded based on artifacts during recordings and lastly two more subjects were excluded based on preset performance criteria during the memory task of <75% (pressing at random could not be excluded). Therefore, 10 lean (f/m = 7/3, age 25.7±1.5 years, BMI 20.9±0.4 kg/m^2^) and 10 obese subjects (f/m = 7/3, age 26.7±1.8 years, BMI 28.8±0.6 kg/m^2^) took part in our investigation. None of the subjects suffered from any severe disease or eating disorder. Volunteers with diabetes mellitus or with a family history of diabetes were excluded at screening, as well as those undergoing treatment for a chronic disease or taking any form of medication. Furthermore, a routine blood analysis was conducted to detect any unknown metabolic or organic diseases. All subjects were normal sighted or had corrected-to-normal vision.

### Study protocol

The placebo and insulin study was conducted on two days in a single-blind randomized cross over study. After 12 hour overnight fast, the experiment was started at 8: 00 am with basal blood sampling and basal MEG measurements. This was followed by intranasal insulin or placebo administration. When insulin levels in the cerebrospinal fluid reached their highest concentrations after 30 minutes [19], the second MEG measurement was performed. Blood was taken every 30 minutes from the beginning of the experiment to determine plasma glucose, insulin and C-peptide levels. The study lasted approximately two hours.

### Nasal spray administration

Nasal spray was administered via spray pumps as previously described [27]. Subjects received either insulin (U100, Insulin Actrapid; Novo Nordisk, Mainz, Germany) or placebo (HOE 31 dilution buffer for H-Insulin; Aventis Pharma, Bad Soden, Germany). Each dosage consisted of 10 IU in a total volume of 0,1 ml and the subjects were administered a totel of 16 doses (160 IU). To ensure optimal absorption, the total dosage was applied in 3 minutes. Both, the insulin and the placebo spray contained cresole so that the subjects could not differentiate between the two sprays.

### Analytical measurements

Plasma glucose was determined using the glucose-oxidase method (YSI, Yellow Springs Instruments, Yellow Springs, CO, USA). Plasma insulin and C-peptide levels were determined with commercial chemiluminescence assays for ADVIA Centaur (Siemens Medical Solutions, Fernwald, Germany).

### Stimulus material

The subject had to perform a one-back visual memory task while inside the MEG. During the task, 64 food and 64 non-food pictures matched for color, size and complexity were presented in a randomized order. The subject was asked to determine whether the current picture belonged to the same object category as the previous one (one-*back* task). If the second picture belonged to the same object category as the first one, the subject pushed the button with their right index finger (“same” category -e.g. food – food: FF, and non-food – non-food: NN). If the picture did not belong to the same object category, the subject pushed the button with their right middle finger (“not-same” category -e.g. food – non-food: FN and non-food – food: NF) (for a more detailed description of the protocol see Stingl et al. [24]). Subjects were excluded from further analysis if the percentage of correct answers was below 75%. Stimulus presentation was controlled with Presentation® (Neurobehavioral Systems, Inc., Albany, CA.).

### MEG data acquisition

MEG signals were recorded using a 275 sensor whole head system (VSM, Medtech, Vancouver, Canada). The continuous recording was filtered off line with a 40 Hz low-pass and a 1 Hz high pass filter and separated into trials of 2100 ms length (from −100 ms to 2000 ms) according to the previous stimulus (conditions FF, FN, NF, NN). The baseline of each trial was defined according to the pre-stimulus interval of 100 ms. All of the trials with eye movement artifacts (detected by threshold criteria) were excluded from further analysis. The trials were averaged for each condition and each subject, and then again averaged for all subjects for every experimental condition. According to our recent study, two different evoked potential components (M1: 100–140 ms, generated in primary visual areas and M2: 140–190 ms, generated in higher visual areas, especially the fusifrom gyrus) showed stronger responses after the presentation of food pictures compared to non-food pictures [24]. The response for the two time windows was quantified by the mean of the root mean square values (RMS) of all channels of the evoked potentials for the specific window and by latency of maximum amplitude of RMS for that component. Furthermore, we showed that there is no difference between conditions for satiated subjects when the previous stimulus was food or non-food [24]. This result was reproduced in the current study and based on this, we combined the conditions FF and NF to the condition “food” and NN and FN to the condition “non-food” for the investigation of the effect of insulin.

### Ethics

The protocol was approved by the local Ethics Committee in Tübingen, Germany and informed written consent was obtained from all participants.

### Statistical analysis

All data are given as unadjusted mean ± SEM. Non-normally distributed parameters were log transformed to approximate normal distribution prior to statistical analysis. MANOVA analysis was used to test for significant differences in metabolic parameters between insulin and placebo experiments with the software package JMP 7.0 (SAS Institute, Cary, NC). Results with p≤0.05 were considered statistically significant.

For the analysis of the behavioral and basal measurement, we used a three-way repeated analysis of variance (ANOVA) with two within factors “preceding stimulus” (levels: food and non-food) and “current stimulus” (levels: food and non-food) and one between factor weight (levels: obese and lean).

The analysis on the insulin effect was preformed for “food” and “non-food” condition. As a measure of insulin action, we calculated the difference for M1 and M2 after insulin application and basal measurement adjusted for the placebo measurement [(insulin 2 – insulin 1) – (placebo 2 – placebo 1)]. A two tailed t-test was performed separately for each condition (food, non-food) and each group (lean, obese) for both periods to determine the effect of insulin. For statistical analysis SPSS 14.0 (SPSS Inc., Illinois; U.S.A.) and Matlab 2007 (The MathWorks, Inc., Natick, USA) were used.

The source activation was calculated for the difference between insulin application and basal measurement adjusted for the placebo measurement [(insulin 2 – insulin 1) – (placebo 2 – placebo 1)] for each subject. For source reconstruction, an empirical Bayesian approach [28]–[30] was used in the time window 100–350 ms (SPM8 http://www.fil.ion.ucl.ac.uk/spm). A standard cortical surface template was transformed to match the fiducials of the MEG data [29]. In all cases, the sensor locations were registered to source space and the gain matrix was computed using a single shell head model. The data covariance was used to estimate the Multiple Sparse Priors maximizing the free-energy approximation to the model-evidence (using automatic relevance determination) [31]. For the second level analysis, we applied a spatial filter of 12 mm. The solutions from individual subjects were grouped and we used a t-test to calculate the statistical parametric map for calculation of areas which showed specific insulin effects. We report all regional activations above the initial significance threshold p<0.05 (FWR corrected).

## Results

### Metabolic parameters

Obese subjects had slightly higher HbA1c as compared to lean subjects (5.5±0.1% vs. 5.4±0.1 in lean). Furthermore, fasting plasma glucose, insulin and C-peptide concentrations were higher in the obese group as compared to the lean group (glucose: p<0.001, insulin: p< = 0.01, C-peptide: p< = 0.001, Table 1). There was no statistical difference in blood glucose levels between the placebo and insulin condition in both the lean and the obese group. After insulin application, insulin levels remained unaltered and C-peptide concentrations did not differ between the insulin and placebo conditions (Table 1).

**Table 1:** Metabolic parameters during MEG experiment (Insulin or placebo spray was given at time = 0 min).

	Placebo	Insulin	Placebo	Insulin	Placebo	Insulin	Placebo	Insulin	Placebo	Insulin	Placebo	Insulin
	Lean subjects		Obese subjects		Lean subjects		Obese subjects		Lean subjects		Obese subjects	
Time (minutes)	Glucose (mmol/l)				Insulin (pmol/l)				C-Peptide (pmol/l)			
−30	4.7±0.1	4.6±0.1	5.2±0.1	5.1±0.1	39±5.6	34±3.9	59±10.3	68±11.9	360±36	336±34	520±53	609±52
0	4.7±0.1	4.8±0.1	5.3±0.2	5.3±0.2	33±5.5	33±5.1	61±11.8	62±11.4	379±53	336±39	554±69	584±62
30	4.4±0.1	4.6±0.1	5.0±0.1	5.0±0.1	33±4.8	41±5.8	70±15.9	88±15.5	345±36	314±38	535±66	595±82
60	4.4±0.1	4.6±0.1	5.1±0.2	5.1±0.1	32±6.8	34±4.8	65±12.1	66±9.1	372±51	289±29	555±67	544±68
p-value (time* insulin/placebo)	0.31		0.76		0.42		0.23		0.19		0.07	

All data are given as mean ± SEM.

Statistical significance between insulin and placebo condition was performed using MANOVA analysis, univariate test and interaction effect.

The p-value time*insulin/placebo shows differences in the curves over time of insulin and placebo.

### Behavioral results for basal measurements

Behavioral data from two basal measurements were combined for each subject. There was a significant main effect for the factors previous and current. The accuracy of the response was higher if the previous picture was food-related (F(1,18) = 37.46, p<0.001). For the current factor, we also found the accuracy to be higher for food pictures compared to non-food pictures (F(1,18) = 14.28, p = 0.0013). For reaction time (RT), the main significant effect was for the factors previous and current. The RT was significantly decreased in conditions where food was the preceding factor (F(1,18) = 136.47, p<0.001) and for conditions where food was the current factor (F(1,18) = 9.28, p = 0.0069) in comparison with non-food. The interaction between preceding and current factor was only statistically significant for accuracy (F = 36.8, p<0.001). There was no significant main effect for the factor weight or interaction effect between weight and any other factor for accuracy or RT.

### MEG data for basal measurements

**Figure 1:**
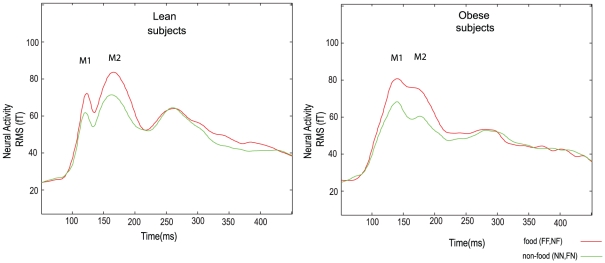
Basal evoked potentials in lean and obese subjects. Time traces of evoked magnetic fields quantified by RMS for food and non-food conditions during the baseline measurement for lean (left) and obese subjects (right). The time point zero indicates the time when the “current” stimulus was shown. For lean and obese subjects, a significant difference in the M1 and M2 components were found for the factor “current” stimulus, e.g. an increase in M1 and M2 for food versus non-food stimulus.

The wave form of the evoked potentials showed two peaks (M1 and M2) within the first 200 ms both in lean and in obese subjects. The main factor current was significant for the RMS values. A significant increase in RMS was observed at both peaks for food compared to non-food in lean and obese subject (M1 - F(1,18) = 36.05, p<0.001 and M2 F(1,18) = 29.75, p<0.001) (Table 2, Fig. 1). None of the other factors or interactions was significant. The RMS values for the M1 and M2 component and the peak amplitude analysis between the lean and obese group were not statistically significant (see Table S1 and Table S2). In addition, no significant main effect or interaction was found for the latency of the M1 and M2 components (Table 2).

**Table 2:** RMS and latency of the components M1 and M2 for the four experimental conditions.

	FF	NF	FN	NN
RMS Lean-M1 (fT)	57±4	60±3	53±3	52±3
RMS Obese-M1 (fT)	64±2	64±2	57±2	55±2
RMS Lean-M2 (fT)	72±3	77±3	63±2	63±2
RMS Obese-M2 (fT)	74±3	77±3	60±2	60±2
Mean Latency Lean-M1 (ms)	124±1	123±1	122±1	122±1
Mean Latency Obese-M1 (ms)	126±2	125±2	124±2	125±2
Mean Latency Lean-M2 (ms)	170±2	172±2	168±1	175±3
Mean Latency Obese-M2 (ms)	171±1	170±1	172±2	171±2

All data are given as mean ± SEM.

fT = femto Tesla, 10^−15^ Tesla.

### MEG results after intranasal insulin application

Neither reaction time nor accuracy of the response to the pictures showed a significant effect related to the insulin application.

**Figure 2:**
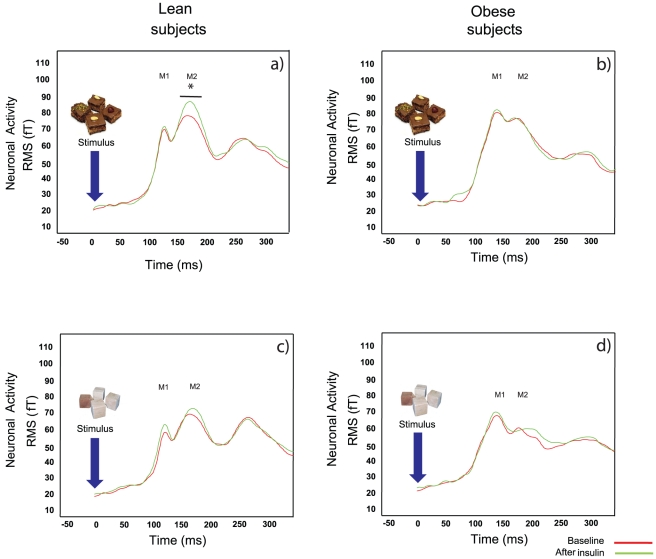
Evoked potentials before and after insulin application. Time traces of evoked magnetic fields quantified by RMS for the measurements before (red line) and after intranasal (green line) insulin application. In the upper row, the response of lean (a) and obese (b) subjects to food stimuli as “current” stimulus are shown. In the lower row, the responses to non-food-pictures are shown (c: lean, d: obese). Only for lean subjects, a statistical significant difference between basal and insulin in the M2 component was found.

The M1 component for food pictures was not affected by insulin. However, insulin led to an increase in the M2 component for food pictures in lean subjects (p = 0.005) but not in obese subjects (Fig. 2). The insulin application did not induce significant changes in neuronal activity for the non-food pictures in lean or obese subjects for both components (Fig. 2).

**Figure 3:**
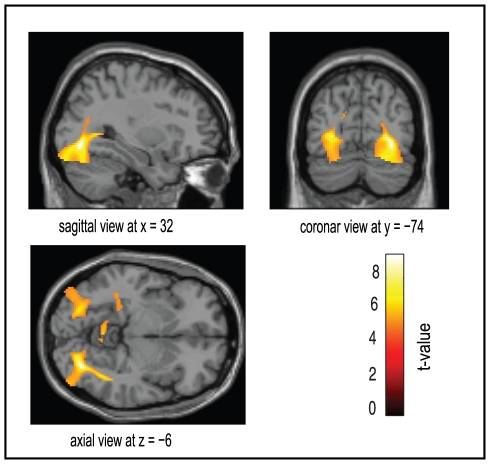
Source analysis of the insulin effect. Brain areas modulated by insulin administration during the working memory task in the food condition. Results are shown for the time period between 100 and 350 ms for all subject rendered onto the surface of a standard anatomical brain volume (Montreal Neurological Institute). All activations are significant at p<0.05 (FWR corrected).

The source analysis revealed significant insulin-mediated changes in a neural network involving visual, temporal and parietal areas with the strongest change in activity in the inferior occipital region (Fig. 3).

## Discussion

Previous studies suggested that the increase in cortical activity during a hyperinsulinemic-euglycemic clamp is prominent in lean subjects whereas this effect was suppressed in obese subjects [14] which indicates insulin resistance in the brain. As it has been demonstrated in various mouse models, insulin resistance in the brain is one factor in the pathogenesis of overeating and obesity [13], [18], [32]–[34]. We therefore evaluated the processing of food pictures by using the MEG technique and its modulation by insulin in lean and obese subjects. Food- and non-food pictures were matched for visual complexity and to account for alterations in the periphery and at the level of the blood brain barrier, insulin was administered intranasally to enter the brain via the olfactory nerve and bulb without affecting metabolic parameters in the periphery [19], [20].

In our study, the increase of the M1 component for food compared to non-food stimuli during the basal measurement replicates the finding for lean, satiated subjects [24]. Furthermore, an equivalent increase was shown in lean and obese subjects after an overnight fast in the current study. The reduction of RT and increase of accuracy, as shown in our previous study [24], is related to the memorization of the preceding food picture. The behavioral measures and the M1 and M2 component of the evoked fields were not statistically different in lean and obese subjects.

Besides the fact that M1 and M2 components are not different at the basal level between lean and obese subjects, we found that food-related brain activity in the visual area was increased in lean subjects following intranasal insulin application, while obese subjects showed no effect and therefore displayed cerebral insulin resistance.

An insulin-mediated increase in cerebral activity in lean subjects was previously described in a study from our group during a hyperinsulinemic-euglycemic clamp with systemic insulin administration [14]. Another support for insulin action in the brain was found in a functional magnetic resonance imaging study with intranasal administration of insulin on a visual recognition task using food and non-food pictures [22]. After insulin administration, there was significantly reduced activity in the presence of food pictures in the right and left fusiform gyrus, the right hippocampus, the right temporal superior cortex and the right frontal middle cortex that might be linked to the termination of food intake.

As insulin did not modulate brain action in lean subjects when non-food pictures were presented [22], we conclude that insulin has a specific effect on brain activity related to identification and categorization of food, which is also confirmed by results at the source level. This might be the reason why other protocols like checkerboard visual tasks, as used in the study from Seaquist et al. [16], were not able to discriminate the effect of insulin on visual evoked potentials.

Source space analysis revealed the largest significant activity change in the inferior occipital areas including the fusiform gyrus in lean but not in obese subjects. This indicates that during this task, mainly perceptional brain areas are activated. In keeping with this, lean subjects showed increased activity by food objects in perceptional visual areas which were significantly attenuated by two days of overfeeding [35]. In that study, it was not reported whether there was a change in insulin sensitivity in these subjects during this intervention; however it can be speculated that overfeeding promotes insulin resistance in the brain to diminish brain activity. Further studies should aim to elucidate the connection of the perceptional effect on reward and cognitive processing that are related to feeding behavior. Based on the work by Figlewitz and Benoit [36] it is suggestive that insulin interacts with feeding related reward areas especially through the midbrain dopamine system which is altered in obese subjects [37].

We want to acknowledge some limitations of the present study. First of all, the number of subjects investigated was rather small. As Hallschmid et al. [20] showed that intranasal insulin reduced body fat in men but not in women, a limitation of our study is that we used more female than male subjects. However, females were shown to have greater prefrontal neuronal responses to food cues compared to males [38]. Furthermore, as demonstrated by Clegg et al. [39], insulin action in the brain is influenced by gonadal hormones. In our study, the time of measurement was not concerted with the menstrual cycle, but in a recent study [40] it was demonstrated that mainly activity in the corticolimbic system is affected by the menstrual cycle during visual food stimulation. In addition, studies reporting gender differences during visual food stimulation demonstrated mainly effects by physiological interactions (satiated versus hungry) or changes in higher processing areas [40]. Based on these studies, we assume that our results are not affected by these factors. Secondly, it would have been an advantage to have accurate insulin levels in the cerebrospinal fluid, however, we were not able to add this procedure to our setting. The dose of insulin was based on a study from Benedict et al. that used 160 IU of intranasal insulin per day to demonstrate significant changes in memory [17], [41] and previous studies detected elevated insulin levels in the cerebrospinal fluid with doses as low as 40 U of insulin intranasally [19]. We chose to not take real food as stimuli of neuronal activation as food-related information is primarily processed by the visual system and it is well established that food pictures differentially activate brain regions compared to non-food pictures [42], [43]. Therefore, stimulation with food pictures is a powerful and easy to handle tool in investigating the mechanisms of food intake.

Taken together, our data show that insulin in the brain potentiates food-related neuronal activity in the components that represent identification and categorization of food pictures in lean subjects, while this was absent in obese. Whether this contributes to overeating and the pathogenesis of obesity needs to be evaluated in further studies.

## Supporting Information

Table S1 Results of the RMS analysis for the M1, M2 components and M1/M2 ratio for the lean and obese group. A two–tailed t-test was used to test for statistical differences between the groups. Data are presented as mean ± SD.
(DOC)

Table S2 Results of the peak amplitude analysis for the M1, M2 components and M1/M2 ratio for the lean and obese group. A two–tailed t-test was used to test for statistical differences between the groups. Data are presented as mean ± SD.
(DOC)
