# Supplementary material for: The Insulin-Mediated Modulation of Visually Evoked Magnetic Fields Is Reduced in Obese Subjects
Source: PLoS One. 2011 May 11;6(5):e19482. doi: 10.1371/journal.pone.0019482 (PMC3092755; doi:10.1371/journal.pone.0019482)
Supplement: Table S1 — Results of the RMS analysis for the M1, M2 components and M1/M2 ratio for the lean and obese group. A two–tailed t-test was used to test for statistical differences between the groups. Data are presented as mean ± SD. (DOC) [file pone.0019482.s001.doc]

**Supporting Information Table 1**

**Results of the RMS analysis for the M1, M2 components and M1/M2 ratio for the lean and obese group. A two–tailed t-test was used to test for statistical differences between the groups. Data are presented as mean ± SD.**

| Components (FF, NF)  FOOD | lean | obese | p |
| --- | --- | --- | --- |
| M1 | 72.4 ± 14 | 80.9 ± 27 | 0.39 |
| M2 | 83.8 ± 42 | 75.94 ± 34 | 0.64 |
| M1/M2 | 0.98 ± 0.32 | 1.12 ± 0.3 | 0.31 |
| Components (NN, FN)  NON FOOD |  |  |  |
| M1 | 61.8 ± 16 | 68.1 ± 25 | 0.49 |
| M2 | 71.3 ± 30 | 60.4 ± 23 | 0.37 |
| M1/M2 | 0.96 ± 0.33 | 1.2 ± 0.49 | 0.188 |

All data are given as mean ± SD.

Methods for the supplementary tables:

In the analysis of RMS values for the M1, M2 components and M1/M2, we combine the RMS values for all channels at the time point defined by peak amplitude on M1 and M2 in combined data sets. In the analysis of peak amplitude, we identified the M1 and M2 components for each individual subjects and extracted peak to peak difference for the component.
